# Frontiers of Innovation and Clinical Application in Endoscopic Endonasal Transsphenoidal Surgery

**DOI:** 10.3390/jcm15041504

**Published:** 2026-02-14

**Authors:** Daisuke Tanioka, Ikuya Natori, Yoichi Morofuji

**Affiliations:** Department of Neurosurgery, School of Medicine, Showa Medical University, 1-5-8 Hatanodai, Shinagawa-ku 142-8555, Tokyo, Japan; natori@med.showa-u.ac.jp (I.N.); youichi.morofuji@showa-u.ac.jp (Y.M.)

**Keywords:** endoscopic endonasal transsphenoidal surgery, pituitary neuroendocrine tumor, skull base surgery, technological innovation, surgical platform, artificial intelligence, neuronavigation, skull base reconstruction

## Abstract

**Background/Objectives**: Endoscopic endonasal transsphenoidal surgery (ETSS) has undergone substantial evolution driven by continuous technological innovations and is increasingly established as a minimally invasive and highly precise approach for the treatment of pituitary neuroendocrine tumors (PitNETs) and selected parasellar lesions. The objective of this review is to summarize the historical development of ETSS and to provide an integrated overview of recent advances shaping contemporary neuroendoscopic surgery. **Methods**: A narrative review of the literature was conducted focusing on key technological and conceptual developments in ETSS, including advances in endoscopic visualization systems, artificial intelligence (AI)-based image analysis, intraoperative navigation, educational support frameworks, and skull base reconstruction techniques. Representative clinical studies and review articles were examined to contextualize current applications and limitations. **Results**: Recent innovations have expanded the functional capabilities of ETSS beyond pituitary surgery alone. Progress in visualization, navigation, and reconstruction techniques has contributed to improved anatomical understanding, surgical safety, and outcome optimization. Furthermore, accumulating clinical evidence supports the selective extension of ETSS indications to complex midline skull base pathologies, including craniopharyngiomas, meningiomas, and chordomas, while emphasizing the importance of appropriate patient selection. **Conclusions**: ETSS has evolved from a single operative technique into an integrated surgical platform supported by technological convergence. Ongoing refinement of visualization, digital assistance, and reconstructive strategies is expected to further enhance safety and precision. This review highlights current trends in ETSS and outlines future directions for innovation and clinical application in neuroendoscopic skull base surgery.

## 1. Introduction

Endoscopic endonasal transsphenoidal surgery (ETSS) has profoundly transformed the surgical management of pituitary neuroendocrine tumors (PitNETs) and parasellar lesions over the past several decades. The systematic adoption of the endoscope as the primary visualization modality in transsphenoidal surgery, first established by Jho et al., marked a paradigm shift from microscopic to endoscopic approaches [1]. Compared with conventional microscopic transsphenoidal surgery, ETSS offers a wider and clearer operative field while remaining less invasive than transcranial approaches (TCA). These characteristics have facilitated deeper anatomical understanding and have contributed to improvements in both the precision and safety of tumor resection.

Importantly, this transition cannot be considered a simple replacement of visualization tools. Rather, it represents a broader conceptual transformation in skull base surgery, namely, a shift from microsurgical to endoscopic surgical thinking. The adoption of endoscopy has permitted direct visualization of anatomical structures that were previously challenging or impossible to observe under the microscope, including the suprasellar region, parasellar compartments, and cavernous sinus [2,3]. Hence, lesions that were once considered beyond the indication of transnasal approaches have become accessible through extended endoscopic endonasal routes. This evolution has redefined surgical anatomy and affected operative strategy, surgical education, and the development of multidisciplinary, team-based practice.

In recent years, the evolution of ETSS has further accelerated through the introduction of high-definition (HD) endoscopic systems, three-dimensional (3D) imaging [4], ultra-HD visualization technologies, including 4K systems [5], advanced intraoperative navigation, and emerging applications of artificial intelligence (AI) in image analysis and intraoperative support [6,7]. Through the convergence of these innovations, ETSS has progressed beyond a single operative technique and now functions as a comprehensive technological platform. These developments support surgeons’ spatial recognition and intraoperative decision-making and help improve reproducibility, standardization, and efficiency in clinical practice and surgical training.

Despite these advances, the rapid expansion of endonasal approaches has raised important questions regarding the nature of the so-called “ETSS revolution.” In particular, whether this revolution should be understood simply as an expansion of surgical reach, or rather as a qualitative transformation in how transsphenoidal surgery is conceptualized, remains unclear.

**Figure 1:**
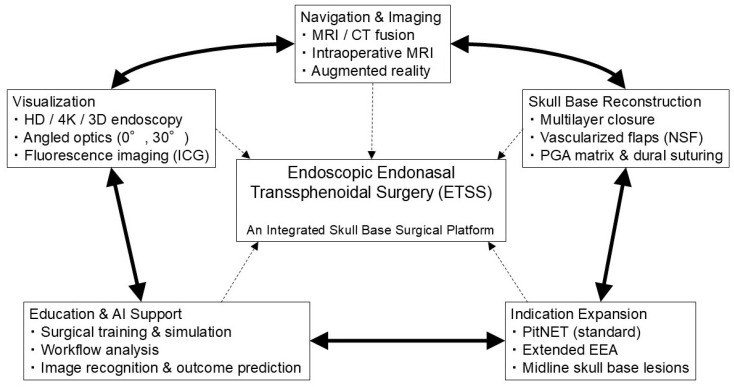
Conceptual framework of the ETSS revolution as an integrated surgical platform. This review conceptualizes endoscopic endonasal transsphenoidal surgery (ETSS) as a paradigm shift from an individual, technique-centered procedure to an integrated skull base surgical platform. The interdependent maturation of multiple domains—including visualization technologies, advanced imaging and navigation systems, skull base reconstruction strategies, and surgical education incorporating AI-based support—has driven this transformation. Together, these elements redefine surgical safety and intraoperative decision-making. Consequently, ETSS represents not merely an expansion of surgical corridors but a qualitative transformation in anatomical understanding, risk management, and functional preservation, thereby supporting the controlled extension of indications beyond pituitary neuroendocrine tumors to selected midline skull base lesions. Bold solid bidirectional arrows indicate reciprocal and co-evolutionary relationships among the major technological domains, whereas dashed arrows represent the integrative contribution of each component to ETSS as a unified surgical platform, as well as feedback effects from ETSS to individual domains.

This review aims to investigate why the evolution of ETSS can be considered a surgical revolution by systematically integrating its historical development, technological innovations, and clinical applications. We further discuss emerging trends in education, training, and AI-assisted surgery, focusing primarily on PitNETs and addressing extended indications, including craniopharyngiomas, meningiomas, and chordomas. Here, we propose a conceptual framework in which ETSS evolution is understood not as a linear accumulation of technical advances but as the establishment of an integrated skull base surgical platform formed through the interaction of multiple interdependent components (Figure 1).

## 2. Materials and Methods

This narrative review comprehensively summarizes the fundamental concepts, technological innovations, clinical applications, and future perspectives of ETSS, incorporating a structured literature search. Literature searches were primarily conducted in PubMed and Web of Science, with combinations of keywords including “endoscopic endonasal transsphenoidal surgery,” “pituitary surgery,” “skull base endoscopy,” “technological innovation,” and “artificial intelligence.” The primary search period was set from approximately 2000 onward, preferentially including original articles, review articles, and technical reports.

This study was not intended to be a systematic review; thus, representative articles were selected based on their clinical and technological relevance and were integrated and interpreted from a specialist perspective. Further, seminal publications predating the defined search period were selectively cited when necessary to describe the historical background and conceptual foundations of ETSS.

## 3. Historical Evolution of ETSS

**From the conceptual origins of transnasal surgery to the establishment of a skull base surgical platform**

ETSS did not abruptly emerge as the result of a single technological breakthrough. Rather, its development represents the gradual refinement of a fundamental surgical concept—namely, achieving minimally invasive access to pituitary and parasellar lesions through a direct midline route—over nearly a century. The historical evolution of ETSS is understood as a stepwise process characterized by three sequential but interrelated phases: (1) transnasal surgical corridor establishment, (2) visualization and illumination technology advancement, and (3) progressive expansion of anatomical regions that are accessible through the endonasal route.

**Figure 2:**
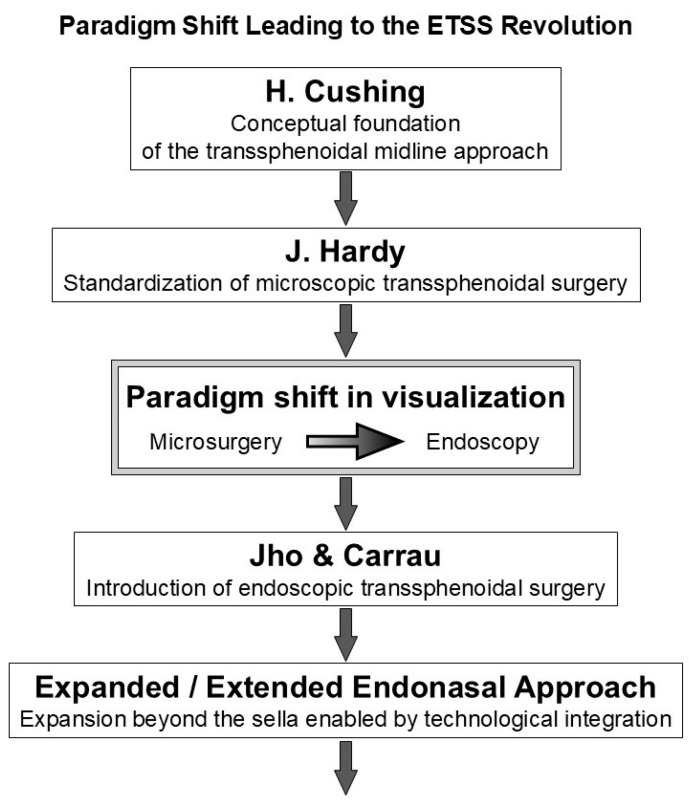
Paradigm shift leading to the ETSS revolution. This schematic demonstrates the historical and conceptual progression underlying the ETSS revolution. Harvey Cushing introduced the fundamental concept of a transsphenoidal midline approach to pituitary lesions, which was later standardized as microscopic transsphenoidal surgery by Jules Hardy. A major paradigm shift was observed with the transition in visualization from microscopy to endoscopy, fundamentally changing anatomical understanding and intraoperative strategy. Building on this shift, Jho and Carrau established endoscopic transsphenoidal surgery, enabling the expansion of surgical indications beyond the sella. The subsequent development of the extended endonasal approach represents the integration of endoscopic visualization with complementary technologies rather than a simple enlargement of the surgical corridor. Vertical arrows indicate the chronological and conceptual progression of surgical development, whereas the horizontal arrow highlights the paradigm shift in visualization from microscopy to endoscopy.

This evolutionary trajectory demonstrates how innovations in surgical approach, optical devices, and anatomical understanding accumulated over time, thereby enabling the transition from early transsphenoidal procedures to modern endoscopic skull base surgery. Figure 2 illustrates the major milestones underlying this transformation, from the early development of transnasal pituitary surgery to contemporary endoscopic endonasal approaches.

### 3.1. Harvey Cushing: The Conceptual Origin of the Transnasal Approach

In the early era of pituitary surgery, Harvey Cushing adopted and advanced the transnasal transsphenoidal approach, originally reported by Schloffer, in response to the substantial invasiveness and high perioperative morbidity of the intracranial approaches that predominated at that time [8]. Recognizing the potential of this route, Cushing further developed it into a systematic therapeutic strategy for pituitary disorders.

This approach was structured based on the anatomical principle that the pituitary gland is located in the midline at the deepest portion of the skull base and, therefore, could be accessed through the shortest and most rational surgical corridor. Through his clinical experience, Cushing clearly articulated the surgical validity and conceptual feasibility of this midline approach [9].

Despite significant technical limitations, his work was highly pioneering in that it established the fundamental idea that pituitary lesions did not inevitably require craniotomy. Concurrently, transnasal surgery was performed under direct naked-eye visualization, without adequate illumination or magnification, leading to severe constraints in operative visibility and maneuverability. Cushing’s efforts clearly revealed that transsphenoidal access represented a legitimate alternative surgical pathway, thereby fundamentally expanding the conceptual options for pituitary surgery [10].

Subsequently, the transnasal approach temporarily fell out of favor among surgeons, including Cushing himself. This decline was largely associated not with intrinsic shortcomings of the approach but rather with the limitations of perioperative management in that era, including the absence of steroid replacement therapy and effective antimicrobial treatment. Consequently, endocrine failure and infectious complications significantly limited surgical outcomes. Importantly, these limitations did not undermine the anatomical rationality or conceptual validity of the transnasal transsphenoidal route [11].

Thus, the underlying surgical philosophy—direct midline access to the pituitary gland through the shortest possible corridor—was preserved, although Cushing’s transnasal surgery was temporarily abandoned. This concept later served as the foundational framework upon which subsequent technological innovations were developed, thereby forming the conceptual substrate for the later introduction of the operating microscope and endoscope.

### 3.2. Jules Hardy: Standardization and Outcome Improvement Through Microscopic Surgery

A major qualitative turning point in transsphenoidal surgery was achieved by introducing the operating microscope by Jules Hardy. The microscope provided stable illumination and magnified visualization, which dramatically improved the accuracy with which pituitary tumors and surrounding anatomical structures could be determined. Hence, transsphenoidal surgery evolved from a highly specialized technique into a standardized and reproducible therapeutic procedure [12].

The establishment of microscopic transsphenoidal surgery substantially improved clinical outcomes in pituitary tumor treatment, with demonstrable gains in hormonal remission rates and reductions in perioperative complications. Concurrently, this success indicated the inherent structural limitations of microscope-based surgery [11].

Microscopic surgery is fundamentally dependent on a linear line-of-sight aligned with the surgeon’s visual axis; thus, the narrow nasal cavity and sphenoid sinus inevitably restrict both visualization and operative maneuverability. These limitations were acceptable for lesions confined to the sella turcica; however, they became major barriers when tumors extended into the suprasellar or parasellar regions or demonstrated complex growth patterns. Thus, Hardy’s technique could not fully overcome the anatomical constraints imposed by microscopic visualization, although it established safety and standardization.

### 3.3. Jho and Carrau: Transformation of Visual Concepts Through Endoscopic Introduction

The transnasal application of neuroendoscopy by Jho, Carrau, and colleagues subsequently addressed the limitations of microscopic surgery [1,13,14]. The endoscope allows the light source and camera to be positioned in proximity to the surgical target; thus, it provides bright and wide visualization even within narrow operative corridors, making it particularly well suited to the endonasal route [13].

Moreover, endoscopy provides panoramic wide-angle visualization and enables close-up and lateral viewing that are not achievable under the microscope. Angled endoscopes enabled surgeons to directly observe anatomical structures in the suprasellar and parasellar regions, as well as areas adjacent to the cavernous sinus, which had previously been challenging to visualize [2,3]. This development represented not only an expansion of the surgical field but also a fundamental transformation in the quality and depth of anatomical information available during surgery.

This shift in visual perception permitted surgical strategies to be reconstructed based not on “how far the surgeon could see,” but on “how far the surgeon could safely manipulate.” Consequently, the introduction of endoscopy served as an essential catalyst for the intrinsic expansion of indications in transnasal surgery [1].

### 3.4. Establishment of ETSS and Its Expansion into Skull Base Surgery

Endonasal endoscopic surgery gradually extended beyond sellar lesions to include suprasellar, parasellar, and midline anterior skull base pathologies, with the maturation of endoscopic technology and the establishment of collaborative surgical teams involving neurosurgeons and otolaryngologists. The concept of the expanded (extended) endonasal approach systematized transsphenoidal corridors based on the craniocaudal and anteroposterior extent of lesions, defining routes, including sellar, parasellar, transplanum–transtuberculum, and transcribriform approaches [15].

ETSS was redefined from a single pituitary surgical technique into an integrated platform for addressing midline ventral skull base lesions through this modular organization of surgical corridors [16]. In particular, accumulating clinical series that involve predominantly suprasellar lesions established the extended endoscopic endonasal transsphenoidal approach as a realistic and reproducible treatment strategy, thereby effectively consolidating the transition from purely sellar surgery toward skull base-oriented practice [17].

Taken together, the historical development of ETSS is considered a continuous technological and conceptual process that originated with changes in visualization devices and progressed through corridor design and skull base-oriented surgical thinking, thereby forming the foundation of modern endoscopic skull base surgery [15,16,17]. Figure 2 summarizes the major milestones of this evolutionary process.

Collectively, this historical progression illustrates that ETSS evolution was not driven by isolated technical breakthroughs but by the gradual integration of surgical concepts, visualization strategies, and anatomical understanding—an essential prerequisite for the establishment of the integrated ETSS platform conceptualized in Figure 1.

## 4. Technological Innovations Driving ETSS

To provide an overview of the technological domains that collectively drove the evolution of ETSS, Table 1 summarizes the major innovations. These advances did not occur in isolation but progressed through the interdependent development of visualization systems, imaging and navigation technologies, skull base reconstruction techniques, surgical instrumentation, and digital or AI-based support. Each component is described in detail in the following sections, with particular emphasis on how its integration has reshaped contemporary ETSS.

### 4.1. Endoscopic Equipment and Visualization

The development and widespread adoption of ETSS have been strongly dependent on both the technological evolution of endoscopic visualization devices—including image resolution, depth perception, and image processing—and the optimization of surgical approaches developed based on endoscopic viewing. Early standardization clarified the concept of using the endoscope as the sole visualization modality, thereby enabling wide-angle and close-up observation of the sellar and parasellar regions and expanding the intraoperative observable field [18,19].

#### 4.1.1. HD, 3D, and Ultra-HD Systems

The introduction of HD endoscopy increased the amount of visual information available for anatomical identification and microsurgical manipulation, thereby establishing HD systems as the standard visual environment for ETSS. However, intrinsic limitations in depth perception associated with two-dimensional (2D) visualization prompted the development of 3D endoscopic systems. Clinical series of purely 3D endoscopic transsphenoidal surgery and comparative studies between 2D and 3D endoscopy have subsequently been reported [4,20,21].

Improvements in depth perception and subjective operability were emphasized in early clinical series using 3D endoscopy [4]. Retrospective comparative studies revealed no significant differences between 2D and 3D systems in operative time, blood loss, length of hospital stay, or early postoperative complications, whereas surgeons reported improved depth perception and reduced cognitive workload when using 3D visualization [20]. Furthermore, preclinical comparative studies that simulate endoscopic skull base surgery indicated that 3D endoscopy may shorten task completion time, reduce technical errors, and improve spatial cognition and hand–eye coordination compared with HD 2D systems [21]. However, these findings were primarily derived from experimental models, and their direct effect on clinical outcomes remains unclear.

More recently, ultra-HD 4K endoscopic systems have been introduced, with several reports describing their application in endoscopic endonasal skull base surgery [22]. Institutional case series comparing HD and 4K visualization in pituitary surgery indicated that the immersive visual information provided by 4K systems improved surgeons’ confidence in intraoperative resection judgment, although definitive outcome superiority remains unclear [23].

#### 4.1.2. Angled Endoscopes and Multiangled Visualization

In ETSS, the use of angled endoscopes in addition to standard 0° optics is a fundamental strategy for exploring blind spots within the sellar and parasellar regions, including areas concealed behind the tumor capsule, diaphragma sellae, or lateral recesses. This concept of “looking around corners” has been described since the early phase of endoscopic transsphenoidal surgery [19].

Beyond 30° endoscopes, higher-angled optics, including 45° and 70° scopes, have been adopted in selected situations to visualize regions that are otherwise inaccessible under direct line-of-sight, particularly in the suprasellar space, parasellar compartments, and along the medial wall of the cavernous sinus. The availability of multiple angled endoscopes has enabled surgeons to dynamically modify viewing direction without changing the surgical corridor, thereby expanding intraoperative anatomical awareness.

In a retrospective series of endoscopic transsphenoidal procedures, Oertel et al. reported that the adjunctive use of angled optics enabled intraoperative identification of residual tumors located in regions not directly visualized with 0° endoscopy, thereby facilitating additional resection in selected cases [24]. Based on postoperative magnetic resonance imaging (MRI)-based assessment of the extent of resection, the authors concluded that angled endoscopy may contribute to improved radicality in endoscopic surgery for sellar lesions.

#### 4.1.3. Fluorescence Imaging and Indocyanine Green (ICG)

Beyond conventional visible-light imaging, fluorescence-based visualization has been explored as a modality to assist in identifying vascular structures and differentiating between tumor and normal tissue. Several studies have investigated the use of ICG fluorescence endoscopy in pituitary surgery, focusing on its potential to visualize tissue perfusion patterns and anatomical relationships [25]. Additional reports have described the applicability, limitations, and image correlations of ICG fluorescence in endoscopic skull base surgery more broadly [26].

However, substantial heterogeneity is observed in administration protocols, imaging timing, and interpretation criteria, and consensus regarding its role as a tumor-specific marker has not been established. Systematic reviews have concluded that the current evidence is limited to small case series and exploratory studies [27]. Accordingly, ICG fluorescence endoscopy has not yet reached routine clinical implementation in pituitary or parasellar surgery and primarily remains an adjunctive or investigational modality.

#### 4.1.4. Emerging Blood Flow-Based Imaging Techniques: Laser Speckle Contrast Imaging

In recent years, laser speckle contrast imaging (LSCI) has been reported as an emerging optical imaging modality for blood flow assessment, primarily in preclinical studies. LSCI enables real-time, non-contact visualization of regional blood flow changes by analyzing laser speckle patterns generated by the motion of red blood cells, and has been widely applied in small-animal models to evaluate cortical perfusion and neurovascular coupling.

Recent experimental studies have demonstrated the feasibility of visualizing cortical hemodynamic dynamics using LSCI in epilepsy models, suggesting its potential utility for functional assessment based on blood flow changes [28]. A distinctive feature of LSCI is its ability to continuously monitor perfusion without the need for exogenous contrast agents, differentiating it conceptually from fluorescence-based imaging and conventional angiographic techniques.

However, to date, there have been no reports of direct application of LSCI in endoscopic endonasal transsphenoidal surgery (ETSS). Several technical limitations remain, including restricted light penetration depth, challenges in maintaining a stable and sufficiently wide surgical field, the confined operative corridor, and the lack of seamless integration with endoscopic platforms and surgical navigation systems. Consequently, in current clinical practice, indocyanine green (ICG) fluorescence imaging remains the most practical and established modality for intraoperative vascular assessment in ETSS.

Nevertheless, from a future-oriented perspective, non-contrast-enhanced, real-time blood flow imaging techniques such as LSCI may serve as adjunctive tools for vascular functional assessment in ETSS, particularly if combined with advances in endoscopic imaging resolution, acquisition speed, and image-processing technologies. Theoretically, the integration of endoscopic visualization with superimposed blood flow maps within the same surgical field, together with quantitative intraoperative perfusion analysis, could provide information beyond simple visualization of vascular anatomy, including early detection of perfusion changes or focal hypoperfusion.

Moreover, if such perfusion-related information were shown to reflect differences in vascular or hemodynamic characteristics between tumor tissue and normal pituitary gland, it could potentially assist in intraoperative discrimination of residual normal pituitary tissue from adenomatous lesions, thereby improving boundary recognition. In this context, the availability of perfusion-based functional information may contribute to more selective tissue handling and avoidance of unnecessary injury to normal pituitary tissue, which could, in turn, be beneficial for preservation of pituitary function.

Furthermore, the recent progress in artificial intelligence-based image analysis raises the possibility that objective pattern recognition of blood flow distributions could complement the surgeon’s subjective intraoperative judgment, supporting tissue differentiation and detection of abnormal perfusion patterns. Although such applications currently remain at a preclinical or conceptual stage, they highlight a potential future direction in which ETSS may further evolve from a mere surgical corridor into a multimodal, information-integrated surgical platform.

#### 4.1.5. Narrow Band Imaging (NBI)

NBI is an image-enhancement technology that emphasizes microvascular patterns using specific light wavelengths. In pituitary surgery, NBI has been investigated mainly for tissue discrimination through visualization of vascular architecture in the normal pituitary gland.

Akutsu et al. reported that characteristic capillary patterns were observed in normal pituitary tissue, whereas adenomas lacked such vascular enhancement, indicating potential utility for gland identification and residual tumor assessment during endoscopic transsphenoidal surgery [29]. However, NBI findings are highly dependent on observation conditions and tumor characteristics, thereby limiting their applicability to selected situations. To date, published studies remain restricted to small clinical series and observational reports, and strong evidence demonstrating improvement in the extent of resection or clinical outcomes is lacking. Consequently, NBI has not been established as a standard visualization modality in ETSS and is currently considered an adjunctive or exploratory technique.

Collectively, advances in endoscopic visualization have not only improved image quality but also fundamentally transformed intraoperative anatomical perception and surgical decision-making, constituting a core component of the integrated ETSS platform conceptualized in Figure 1.

### 4.2. Advanced Neuroimaging and Navigation

Neuronavigation was introduced to resolve the inherent limitations of purely endoscopic anatomical orientation by enabling real-time reference to preoperative imaging data. This capability has become particularly crucial in complex endoscopic transsphenoidal surgery, where reliable intraoperative spatial awareness is essential for safe and precise manipulation.

Currently, intraoperative navigation is increasingly considered a standard adjunctive technology in ETSS. In pituitary surgery, navigation systems that integrate preoperative MRI with intraoperative computed tomography (CT) have been widely adopted in clinical practice. Linsler et al. reported a navigation workflow in which preoperative MRI datasets were registered and fused with intraoperative CT images, thereby enabling 3D stereotactic guidance during surgery. In their clinical experience, this approach demonstrated high navigational accuracy even in the presence of distorted anatomical landmarks, with target registration errors within the submillimeter range under specific conditions [30].

Intraoperative MRI (iMRI) has been investigated as a modality for real-time assessment of residual tumor and immediate surgical feedback. Multiple clinical studies have indicated that iMRI may facilitate residual lesion detection and support intraoperative decision-making, thereby potentially contributing to higher rates of gross total resection (GTR) and improved postoperative visual outcome prediction. In a comprehensive review, Buchfelder et al. revealed that iMRI improves residual tumor visualization and safety assessment in pituitary surgery, while highlighting substantial limitations associated with infrastructure requirements, prolonged operative time, and increased financial burden [31].

More recently, navigation systems based on intraoperative CT with automated registration have been combined with augmented reality (AR) approaches. Several clinical and experimental studies have reported improvements in usability and navigational precision, with target registration errors on the order of <1 mm under controlled settings [32]. However, such performance remains dependent on image quality, registration methodology, and intraoperative conditions.

Despite these advances, intraoperative navigation with MRI/CT fusion in endoscopic transsphenoidal surgery has limitations. Challenges involve imperfect image registration, insufficient compensation for dynamic anatomical changes, including brain shift, and substantial technical, logistical, and cost-related demands. Accordingly, navigation systems significantly improve anatomical orientation and intraoperative decision-making; however, their broader implementation and ultimate clinical effect remain constrained by these practical considerations [31].

Collectively, neuroimaging and navigation advances have shifted intraoperative decision-making from experience-based estimation toward image-supported spatial reasoning, demonstrating a key functional component of the integrated ETSS platform illustrated in Figure 1.

### 4.3. AI-Based Image Analysis and Surgical Support

#### 4.3.1. Segmentation and Intraoperative Image Understanding

Automated recognition and segmentation of intraoperative anatomical structures and tumor regions is one of the most actively developing areas of AI implementation in ETSS. Recent studies have reported deep learning-based analysis of intraoperative endoscopic video, frequently referred to as “videomics,” which enables automatic extraction of pituitary adenoma regions and indicates a potential role in assisting intraoperative tumor boundary recognition [33]. Further, several investigations have revealed AI-based identification of anatomical landmarks from endoscopic pituitary surgery videos, thereby positioning such systems as adjunctive tools to support surgeons’ intraoperative orientation [34].

Beyond static anatomical labeling, automated recognition of surgical workflow from operative videos has been investigated. These approaches can be used not only for real-time intraoperative support but also for postoperative quality assessment, structured data generation, and educational feedback [35]. However, these models are highly sensitive to domain shifts originating from interinstitutional variability in endoscopic systems, white balance, bleeding conditions, lens contamination, and surgical style. Consequently, external validation and safety-oriented system design—including appropriate visualization strategies and fail-safe mechanisms to address potential misclassification—are essential prerequisites for clinical implementation [34,35].

Altogether, advances in intraoperative image understanding have established a technical foundation for broader AI applications in ETSS. Grounded on this foundation, AI-based methodologies have been extended beyond real-time support toward perioperative decision support systems. In particular, predictive models that integrate preoperative imaging features, intraoperative findings, and early postoperative data have been investigated to estimate postoperative hormonal remission or the need for adjuvant therapy in pituitary disorders, including acromegaly [35]. These approaches conceptually depend on accurate segmentation and structured representation of tumor extent and anatomical relationships as upstream components. However, despite promising results from individual studies, systematic reviews have highlighted substantial heterogeneity in dataset size, outcome definitions, and external validation availability, indicating that high performance in single-center studies does not necessarily translate into broad clinical applicability [36].

#### 4.3.2. Education, Simulation, and Skill Assessment

From an educational perspective, the steep learning curve associated with ETSS has driven a longstanding interest in simulation-based training. Early development of virtual simulators for ETSS demonstrated the feasibility of providing structured training environments for skill acquisition [37]. Subsequent reviews of virtual reality (VR) simulators in neuroendoscopy have summarized technological progress as well as persistent challenges associated with realism, haptic feedback, and validation methodology [38].

Systematic reviews that focus specifically on ETSS training models have further investigated a wide range of platforms, including cadaveric dissection, 3D printed models, and VR-based simulation systems [39]. Further, validation studies of dedicated training boxes have assessed face, content, and construct validity, frequently in combination with objective assessment tools, including the Objective Structured Assessment of Technical Skills (OSATS), thereby developing frameworks for structured skill evaluation [40].

Regarding near-future implementation, two converging directions appear particularly relevant: (i) automated analysis of surgical videos to identify procedural phases, technical maneuvers, and potential errors, which may be associated with personalized educational feedback systems [7]; and (ii) real-time recognition of anatomical landmarks that could be repurposed as guidance tools during simulation training through augmented reality-based visualization [34]. These developments may evolve by integrating existing simulation platforms rather than replacing current educational paradigms.

Collectively, AI-based image analysis and educational support systems represent a natural extension of the integrated ETSS platform, thereby reinforcing its transition from experience-dependent surgery toward data-driven, standardized, and reproducible practice, as conceptualized in Figure 1.

## 5. Innovative Skull Base Reconstruction

**Safety enhancement and complication avoidance in ETSS**

In ETSS, postoperative cerebrospinal fluid (CSF) leak represents one of the most critical complications, as it may require reoperation or CSF diversion and serves as a major risk factor for intracranial infection, including meningitis. Accordingly, skull base reconstruction advances have become a central determinant of surgical safety and form the foundation supporting the expansion of endonasal indications. Clinical series that analyze consecutive cases have demonstrated that CSF leak and infectious complications continue to occur at a measurable frequency after expanded endoscopic endonasal surgery, thereby emphasizing the importance of reconstruction-focused risk analysis [41].

### 5.1. Reduction in Postoperative CSF Leak and Prevention of Meningitis

The size of dural defects and the volume of CSF leakage—particularly high-flow leaks—increased accordingly, as the surgical reach of ETSS expanded from the sellar region to the suprasellar space, clivus, and anterior skull base. Under these conditions, reconstruction strategies relying solely on free grafts, including fat or fascia, often proved insufficient and appeared as a major bottleneck limiting safe indication expansion. Hence, skull base reconstruction underwent a qualitative transformation by adopting multilayer techniques and introducing vascularized tissues.

#### 5.1.1. Multilayer Reconstruction and Vascularized Flaps

Multilayer reconstruction is based on a design concept in which the intracranial (inlay) and nasal (onlay) components serve complementary roles, frequently combined with mechanical buttressing and sealing materials to achieve watertight closure. Within this framework, the introduction of vascularized flaps—most notably the pedicled nasoseptal flap described by Hadad and Bassagasteguy—marked a pivotal advance that substantially improved reconstruction success rates and changed the complication profile of expanded endonasal skull base surgery. Hadad et al. reported a clinical series demonstrating the effectiveness of the vascularized nasoseptal flap for skull base defect reconstruction [42].

#### 5.1.2. Learning Curve and Procedural Standardization

Reconstruction outcomes depend not only on materials and techniques but also on institutional and team-based experience. Early phases of indication expansion were usually associated with higher rates of reconstruction failure, subsequent standardization of defect classification (based on leak flow, defect geometry, and extent of arachnoid violation), algorithm-based selection of reconstruction strategies, standardized flap elevation techniques, and optimized postoperative management—including lumbar drainage indications and nasal packing strategies—have collectively contributed to CSF leak rate reduction at the institutional level [43].

### 5.2. Introduction of Absorbable Dural Substitutes

In addition to traditional autologous materials, including fat and fascia, the introduction of absorbable dural substitutes has expanded reconstruction options in endoscopic skull base surgery. When employed as inlay components, these materials enable multilayer reconstruction while avoiding donor-site morbidity. Clinical case series using collagen matrix grafts for endoscopic skull base reconstruction have reported acceptable postoperative CSF leak and infection rates, with no evidence of delayed reconstruction failure [44]. These findings support the clinical acceptance of absorbable dural substitutes as complementary materials improving reconstruction safety.

More recently, absorbable matrices composed of polyglycolic acid (PGA) nonwoven fabric have been introduced for skull base reconstruction. Tanioka et al. reported the application of a reconstruction strategy that combines dural suturing with a PGA matrix (Durawave™) in endoscopic endonasal surgery, demonstrating a significantly lower postoperative CSF leak rate compared with conventional fat packing techniques [45]. This approach highlights anatomical dural closure supplemented by absorbable materials, thereby minimizing autologous tissue harvesting while aiming for reliable watertight repair.

### 5.3. Dural Suturing Techniques at the Sellar Floor

In addition to coverage and packing-based reconstruction, direct dural suturing has appeared as an alternative reconstructive option that targets the leak point itself. In endoscopic transsphenoidal surgery, suturing of dural incision edges after tumor removal—particularly in cases with limited CSF leakage—has been a feasible technique [46].

This strategy is conceptually based on direct anatomical restoration rather than reliance on bulk grafting materials and represents a rational option for selected cases with localized dural defects. The introduction of dural suturing underscores that reconstruction safety improvements have been driven not only by material innovation but also by the refinement of surgical technique and operative precision.

### 5.4. Prevention of Internal Carotid Artery (ICA) Injury Through Imaging and Navigation

ICA injury remains a rare but potentially catastrophic complication in expanded ETSS. From a safety perspective, meticulous preoperative planning and accurate intraoperative localization are essential, supported by navigation systems, a detailed understanding of vascular anatomy, and preparedness for endovascular intervention.

Large retrospective institutional reviews have reported the incidence, injury patterns, and clinical outcomes associated with ICA injury during endonasal surgery [47]. Further, comprehensive reviews that address prevention and management strategies have highlighted the importance of protocol-based preparedness, including team coordination, availability of dedicated devices, and immediate endovascular support [48].

Collectively, these advances have permitted a transition from merely “achieving access” toward maintaining complications within controllable limits. In this context, skull base reconstruction and safety-oriented imaging strategies constitute key elements underlying the qualitative transformation of ETSS, whereby the concept of surgical revolution is increasingly defined by improved safety rather than expanded reach alone [41,47,48].

Together, innovations in skull base reconstruction and safety-oriented surgical design have helped develop the structural foundation that enables indication expansion to proceed within controlled risk boundaries, thereby forming a critical safety pillar of the integrated ETSS platform conceptualized in Figure 1.

## 6. Clinical Applications and Expansion of Indications

### 6.1. Pituitary Neuroendocrine Tumors

ETSS has been extensively adopted as a primary surgical approach for PitNETs, and a growing body of comparative evidence against microscopic transsphenoidal surgery has accumulated. Systematic reviews and meta-analyses of pituitary adenoma surgery have generally indicated that endoscopic approaches may achieve higher GTR and lower certain nasal complication rates (e.g., septal perforation) compared with microscopic surgery, whereas CSF leak rates and major complications are frequently reported to be broadly comparable between techniques. However, the majority of available studies are retrospective, and the need for higher-quality prospective evidence has repeatedly been emphasized [49,50].

The clinical significance of ETSS for functioning PitNETs is often framed less as an “expansion of indications” and more as maturation into a standard-of-care procedure. Several reports present surgical outcomes (e.g., biochemical remission) alongside safety profiles (complications), while acknowledging that results are affected by tumor subtype, extension pattern, and institutional experience.

Remission outcomes differ by functional subtype, and disease-specific evidence is particularly relevant when discussing remission. Multiple studies have reported improved remission rates for growth hormone (GH)-secreting adenomas (acromegaly) after the adoption of endoscopic surgery compared with the microscopic era. Large cohort analyses have identified an endoscopic approach as an independent predictor of long-term remission when compared with microscopic surgery (odds ratio: 2.8, *p* = 0.001) [51]. Further, consistent trends have been reported in systematic reviews, in which remission rates for macroadenomas were higher in endoscopic cohorts than in microscopic cohorts (60% vs. 46.9%) [52].

One proposed explanatory mechanism is the improved ability of endoscopy to support the identification of the pseudocapsule plane between the tumor and normal pituitary tissue, thereby facilitating extracapsular dissection [53]. This technique has been associated with reduced subcapsular remnants and is a potential contributor to improved biochemical remission in macroadenomas. In this context, ETSS represents not only wider visualization but also the maturation of anatomically precise surgical techniques that leverage endoscopic optical advantages.

Systematic reviews and meta-analyses for Cushing disease have likewise compared outcomes between ETSS and microscopic surgery. Overall remission rates have ranged from approximately 70% to 80% across eras, and consistent superiority of one technique over the other has not been clearly demonstrated. Further, heterogeneity in the definitions of remission and recurrence has been recognized as a major factor affecting comparability and interpretability across studies [54,55].

Regarding complications, institutional experience and learning curve effects play a crucial role in identifying surgical safety. Complication profiles are frequently discussed in large single-center series that also incorporate learning curve effects and refine reconstruction and perioperative management. Analyses of large endoscopic endonasal series, including expanded approaches, reported detailed complication frequencies and emphasized “lessons learned,” thereby highlighting the importance of standardized technique, reconstruction strategies, and team-based workflows in maintaining safety as procedural complexity increases [56].

### 6.2. Extended Indications

#### 6.2.1. Patient Selection and Surgical Boundaries

When discussing extended indications, ETSS should not be regarded as a universally applicable approach or expanded without limitation. Rather, an optimal surgical strategy needs a comprehensive assessment of tumor morphology and extension patterns, relationships with surrounding neurovascular structures, goals of functional preservation, and anticipated risks of complications. Based on these factors, individualized decision-making is required, which may appropriately include TCA or combined endonasal–transcranial strategies in selected cases.

Contemporary clinical series of large and giant pituitary adenomas increasingly focus not only on surgical outcomes achieved with EETS, but also on predictive factors for the extent of resection derived from tumor configuration and growth patterns. Such analyses have helped clarify the practical boundaries of endonasal-only surgery and identify situations in which alternative or complementary TCA should be considered [57,58]. TCA remains essential for tumors with marked lateral extension, extensive neurovascular encasement, or complex dumbbell-shaped growth patterns, even in the modern era of advanced endoscopic techniques [59,60]. Therefore, combined endonasal–transcranial surgery has been positioned as a rational strategy in selected cases to achieve an optimal balance between maximal safe resection and procedural safety [61].

Altogether, the expansion of ETSS indications should not be interpreted as an unlimited anatomical reach but rather as a process of refined patient selection guided by tumor characteristics and clearly recognized surgical boundaries.

#### 6.2.2. Craniopharyngioma

A comparison between TCA and expanded endoscopic endonasal approaches (EEA) is clinically unavoidable for craniopharyngiomas. Recent comparative series have indicated that both approaches can aim for radical resection and durable tumor control; however, outcomes associated with visual function, hypothalamic-related morbidity, endocrine dysfunction, and CSF leak differ depending on tumor subtype and growth pattern and cannot be summarized by a simple “superior/inferior” conclusion [62]. Synthesizing these data, the surgical strategy for craniopharyngioma should be individualized according to tumor location and direction of extension, degree of adhesion to surrounding structures, and the anatomical relationship to perforators and the hypothalamus [62,63]. Accordingly, ETSS represents an important surgical option for selected cases of craniopharyngioma, whereas careful patient selection remains essential, particularly in the presence of extensive hypothalamic involvement or complex tumor-adhesion patterns.

#### 6.2.3. Meningioma

Comparative series restricted to anatomically “equipoise” cases have reported differences in the extent of resection, visual outcomes, brain manipulation, and complication profiles between transcranial and endonasal approaches for tuberculum sellae to suprasellar meningiomas (including planum sphenoidale) [64]. Meta-analyses including anterior skull base meningiomas (olfactory groove and tuberculum sellae) revealed that endonasal endoscopic approaches have not consistently demonstrated overall superiority over microscopic transcranial surgery. Some reports describe advantages, including visual improvement, whereas increased risks of complications—including CSF leak—have also been observed [65]. Larger clinical series in olfactory groove meningiomas have similarly reported favorable outcomes while explicitly identifying limiting factors that affect radical resection and complication risk, including tumor size, calcification, and lateral/anterior dural extension [66].

These findings indicate that endonasal endoscopic surgery for anterior skull base and parasellar meningiomas is most compelling in selected midline lesions where direct inferior access may support visual preservation. Conversely, alternative approaches—including transcranial routes—are frequently recommended for cases with optic nerve lateral extension, substantial encasement of major vessels (ICA/ACA), involvement of the anterior clinoid process, or participation of the cavernous sinus [67,68]. Multi-institutional analyses and scoring studies further reinforce the need for imaging-based preoperative selection, emphasizing that endonasal indications should be carefully defined rather than indiscriminately expanded [69]. Accordingly, ETSS represents a valuable option for selected midline skull base meningiomas, whereas careful patient selection remains essential, particularly in cases with lateral extension beyond the optic canal or significant vascular encasement.

#### 6.2.4. Chordoma

The EEA for clival chordomas provides a rational direct corridor to midline clival pathology and has been reported in systematic analyses and single-center series as a potentially advantageous option in terms of the extent of resection and perioperative morbidity compared with traditional open skull base approaches [70,71]. However, chordomas are locally aggressive and prone to recurrence; thus, tumor control is rarely achieved by surgery alone. Most reports emphasize the importance of multidisciplinary strategies, including adjuvant radiotherapy, while pursuing maximal safe resection [70,71].

The EEA series has also reported reconstruction-related complications, including CSF leak, at nonnegligible frequencies. Accordingly, careful indication selection informed by lateral extension and neurovascular relationships, together with robust multilayer reconstruction, remains central to achieving favorable outcomes [70,72]. Therefore, ETSS represents a rational primary approach for midline clival chordomas, whereas long-term tumor control requires recognition of its limitations and integration of adjuvant therapies within a multidisciplinary treatment strategy. Table 2 presents a pathology-oriented overview of indication boundaries.

Across both PitNETs and extended skull base pathologies, the clinical application of ETSS can be viewed as a pathology-specific process of patient selection supported by integrated platform capabilities—including visualization, navigation, reconstruction, and team-based workflows—as outlined in Figure 1.

## 7. Limitations and Future Directions

### 7.1. Current Limitations of Endoscopic Endonasal Surgery

ETSS/EEA should not be simplistically regarded as a universally safe approach that can be expanded without constraint, despite substantial advances in visualization, surgical corridors, and reconstruction techniques. In particular, EEAs are frequently associated with larger dural defects, increased reconstructive complexity, and a heightened risk of catastrophic complications, including vascular injury. As procedural complexity increases, the overall surgical burden and reconstructive demands may substantially rise, despite employing a minimally invasive corridor. Large single-center series have demonstrated that complex intradural manipulation and specific pathological entities are associated with higher serious complication rates [56]. Further, although rare, ICA injury remains a realistic and potentially fatal complication, underscoring the need for continuous risk awareness and preparedness, particularly in extended approaches [47].

#### 7.1.1. Evidence Gaps and Limitations of Comparative Data

The relative advantages and disadvantages of ETSS compared with conventional approaches—including microscopic transsphenoidal surgery and transcranial surgery—vary considerably depending on pathology, anatomical location, and reconstructive conditions. However, the current evidence base remains dominated by retrospective studies, with limited availability of prospective or randomized comparative trials. For instance, meta-analyses of pituitary adenoma surgery consistently emphasize that most included studies are retrospective and explicitly call for future large-scale prospective investigations [50]. Similarly, available meta-analyses in anterior skull base meningiomas have not demonstrated uniform superiority of endonasal approaches over transcranial surgery, particularly when considering complication profiles, including CSF leak and vascular injury [65]. These findings collectively indicate that careful indication assessment—rather than technical preference—is central to outcome optimization.

#### 7.1.2. Learning Curve and Institutional Dependency

Institutional experience, multidisciplinary collaboration, and procedural standardization strongly affected clinical outcomes after ETSS/EEA. Several studies have demonstrated that increasing surgical experience is associated with decreases in operative time and intraoperative CSF leak, demonstrating a pronounced learning curve effect [73]. Importantly, higher complexity cases frequently require a substantially longer learning period, thereby limiting the applicability of simplified case-number thresholds for proficiency [74]. Reconstruction outcomes similarly show experience dependency, as early-phase series have reported higher postoperative CSF leak rates during the initial adoption of vascularized flap techniques [43]. Furthermore, volume–outcome relationships have been reported, with national registry analyses revealing lower reoperation rates, hemorrhagic complications, and prolonged hospitalization in high-volume centers [75]. These findings indicate challenges in generalizing outcomes across institutions with heterogeneous case volumes and training environments.

#### 7.1.3. Cost, Accessibility, and Implementation Constraints

Advanced technologies, including high-precision neuronavigation, intraoperative CT or MRI, augmented reality-based guidance systems, as well as ultrasound imaging and photoacoustic techniques, provide potential benefits in anatomical orientation and intraoperative decision-making. However, equipment cost, workflow burden, staffing requirements, and institutional infrastructure limit their widespread implementation. For instance, intraoperative MRI has well-recognized advantages as well as substantial logistical and economic limitations that restrict its availability to selected centers [31]. Similarly, augmented-reality-assisted navigation systems may increase preparation time and workflow complexity, thereby limiting routine adoption [32].

#### 7.1.4. Limitations of Current AI-Based Applications

AI-based image analysis and surgical support systems in ETSS have demonstrated promising proof of concept results, particularly in intraoperative image understanding and anatomical landmark recognition. However, most studies remain limited by small dataset sizes and single-center development, thereby raising concerns regarding external validity and generalizability [6]. Systematic evaluations of machine learning-based outcome prediction models in pituitary surgery have identified deficiencies in reporting quality, lack of external validation, and insufficient prospective assessment [36]. Accordingly, AI and advanced navigation technologies should not be viewed as enablers of unrestricted indication expansion but rather as tools that require rigorous validation within clearly defined safety frameworks.

### 7.2. Future Directions Toward Boundary-Aware Implementation

Future progress in ETSS should not be driven by further anatomical expansion alone but also by continuous refinement of indication boundaries. Optimal surgical strategy requires the integration of pathological characteristics, tumor location, neurovascular relationships, anticipated dural defect size, reconstructive requirements, and institutional experience when selecting from endonasal, transcranial, or combined approaches [65]. Therefore, ETSS should be conceptualized not as a competition of surgical reach but as a strategy aimed at maximizing safety and functional preservation through the precise recognition of anatomical and technical limits.

Prospective multicenter data collection is warranted to advance beyond the current evidence structure dominated by retrospective single-center studies. Long-term outcomes—such as visual function, endocrine status, quality of life, and delayed complications—should be evaluated using standardized definitions and reporting frameworks in addition to traditional endpoints, including extent of resection and tumor control [50].

Considering the strong influence of learning curves on surgical safety and reproducibility, structured educational systems tailored to procedural complexity are also required. These include staged training models, standardized reconstruction protocols, and objective assessment of team-based readiness—all aimed at mitigating experience-dependent variability [43,73,74]. Moreover, low-frequency but potentially catastrophic complications, including ICA injury, should be addressed through system-level risk mitigation strategies encompassing protocols, simulation-based training, and institutional backup frameworks rather than relying on individual expertise alone.

Finally, not only technical feasibility but also demonstrated clinical benefit, cost-effectiveness, and accessibility should guide the future integration of advanced technologies—including intraoperative imaging, AR, and AI-based support. Rigorous evaluation of these technologies in real-world clinical settings is essential to ensure that innovation contributes meaningfully to patient safety rather than increasing complexity without proportional benefit [31,32,36].

## 8. Conclusions

The revolution of ETSS should be understood not merely as a consequence of improved visualization or expanded surgical corridors, but rather as a fundamental transformation of transsphenoidal surgery into an integrated skull base surgical platform. The coordinated maturation of multiple interdependent components—including endoscopic visualization, anatomical understanding, reconstruction strategies, image-guided technologies, and team-based surgical practice—has driven this transformation.

Through this platform-based evolution, ETSS has enabled qualitative improvements in surgical safety, intraoperative decision-making, and functional preservation rather than a simple extension of technical reach. Therefore, the clinical impact of ETSS lies not in how far surgeons can anatomically reach, but in how reliably complex pathology can be addressed within clearly defined safety boundaries.

Surgical invasiveness, reconstructive demands, and risk profiles also escalate accordingly, as endonasal procedures increase in complexity. These realities emphasize the necessity of boundary-aware patient selection, standardized training pathways, and robust safety frameworks that extend beyond individual technical proficiency.

Therefore, future ETSS advancement should prioritize evidence-based refinement of indication criteria, reproducible implementation across institutions, and rigorous evaluation of emerging technologies—including navigation systems, intraoperative imaging, and AI-based support—within clinically meaningful and ethically grounded frameworks. Hence, ETSS should be viewed not as an ever-expanding technique but as a continuously evolving surgical platform whose true value relies on the integration of innovation with disciplined limitation.

Consistent with the platform model illustrated in Figure 1, the enduring significance of ETSS depends on its capacity to harmonize technological progress with anatomical respect, surgical judgment, and patient-centered outcome optimization.

## Figures and Tables

**Table 1:** Technological innovations contribute to the evolution of endoscopic endonasal transsphenoidal surgery (ETSS).

Category	Technology	Clinical Relevance
Visualization systems	High-definition endoscopy (HD, 4K)	Improved image resolution and brightness, enabling precise anatomical recognition and safer tumor dissection.
	Three-dimensional (3D) endoscopy	Provides stereoscopic depth perception, improving hand–eye coordination and reducing the learning curve.
	Angled endoscopes (30°, 45°, 70°)	Allows visualization beyond the straight surgical corridor, facilitating access to suprasellar and parasellar regions.
	Narrow band imaging (NBI)	Enhances mucosal and vascular patterns, potentially supporting intraoperative tissue discrimination.
Fluorescence imaging	Indocyanine green (ICG) fluorescence	Real-time visualization of vascular structures and flap perfusion, improving safety during ICA dissection and skull base reconstruction.
	Laser speckle contrast imaging (LSCI)	Preclinical, non-contrast-based visualization of regional blood flow dynamics, with potential future applicability for functional vascular assessment and tissue discrimination.
Neuroimaging and navigation	Neuronavigation systems	Provides real-time anatomical localization based on preoperative imaging, improving spatial orientation and procedural safety.
	MRI/CT image fusion	Integrates bony and soft-tissue information for accurate understanding of complex skull base anatomy.
	Intraoperative imaging (iMRI/iCT)	Updates anatomical information intraoperatively, supporting detection of residual tumor and resection control.
Skull base reconstruction	Multilayer reconstruction technique	Combines inlay and onlay grafts with sealant techniques, significantly reducing postoperative CSF leakage.
	Vascularized pedicled nasoseptal flap	Provides robust reconstruction of large skull base defects and represents the standard method for high-flow CSF leak prevention.
	Absorbable artificial dural substitutes	Allows flexible inlay reconstruction using collagen- or PGA-based matrices while avoiding donor-site morbidity.
Surgical instrumentation	Dedicated endoscopic instruments	Slim, curved, and long-shaft instruments optimized for the nasal corridor improve maneuverability and surgical precision.
Digital and AI-based support	AI-based image segmentation	Automated delineation of tumors and critical structures supports preoperative planning and anatomical understanding.
	Outcome prediction models	Integration of imaging and clinical variables assists perioperative decision-making and risk stratification.
Education and training	Simulation and virtual training systems	Virtual reality and model-based environments support standardized education and improvement of surgical safety.

This table summarizes key technological innovations that have driven the evolution of endoscopic endonasal transsphenoidal surgery (ETSS). These developments span multiple domains, including visualization systems, fluorescence imaging, neuroimaging and navigation, skull base reconstruction, surgical instrumentation, and digital or AI-based support. Collectively, they have enhanced anatomical understanding and surgical safety, thereby supporting the establishment of ETSS as a comprehensive skull base surgical platform.

**Table 2:** Expanded indications and recognized surgical boundaries of endoscopic endonasal transsphenoidal surgery.

Pathology	ETSS-Related Clinical Role	Major Limitations/Boundaries	Key Implication
PitNET	Established as the standard surgical approach with reliable resection outcomes.	Evidence mainly retrospective; outcomes influenced by tumor extension and surgical experience.	Represents maturation of the surgical platform rather than simple indication expansion.
Acromegaly	Improved remission in selected macroadenomas through enhanced visualization and extracapsular dissection.	Invasive growth and cavernous sinus extension limit biochemical cure.	Endoscopic advantage is anatomical, not universal.
Cushing disease	Comparable remission rates to microscopic surgery with improved visualization.	No consistent improvement in remission; heterogeneous definitions among studies.	Benefit lies in precision rather than higher cure rates.
Large/giant PitNET	Selected tumors amenable to endonasal resection.	Marked lateral extension, dumbbell configuration, and vascular encasement.	Alternative or combined transcranial strategies should be considered.
Craniopharyngioma	Effective midline access with potential benefit for visual outcomes.	Hypothalamic involvement and adhesion pattern strongly influence results.	Individualized approach selection is essential.
Meningioma (midline)	Favorable optic nerve decompression in selected tuberculum and planum lesions.	Lateral optic canal invasion and vascular encasement restrict indication.	Strict anatomical selection is mandatory.
Chordoma (clival)	Rational midline corridor enables maximal safe resection.	Local invasiveness and CSF leak risk preclude surgical cure by surgery alone.	ETSS should be integrated into a multimodal treatment strategy.
Overall concept	Broader applicability to selected midline skull base lesions.	Persistent anatomical and biological boundaries remain.	Expansion should be strategy-driven, not reach-driven.

This table outlines representative clinical domains in which ETSS has expanded its applicability, together with key anatomical, biological, and strategic factors that define appropriate patient selection. The information presented emphasizes that the expansion of indications should be understood as the refinement of surgical strategy within established boundaries, rather than an unrestricted extension of surgical reach.

## Data Availability

No new data were created or analyzed in this study. Data sharing is not applicable.

## Abbreviations

The following abbreviations are used in this manuscript:

AI	Artificial intelligence
AR	Augmented reality
CSF	Cerebrospinal fluid
CT	Computed tomography
EEA	Expanded endoscopic endonasal approach
ETSS	Endoscopic endonasal transsphenoidal surgery
GTR	Gross total resection
HD	High definition
ICA	Internal carotid artery
iCT	Intraoperative computed tomography
iMRI	Intraoperative magnetic resonance imaging
MRI	Magnetic resonance imaging
OSATS	Objective Structured Assessment of Technical Skills
PitNET	Pituitary neuroendocrine tumor
VR	Virtual reality
